# Combination of data-driven models and interpolation technique to develop of PM10 map for Hanoi, Vietnam

**DOI:** 10.1038/s41598-020-75547-y

**Published:** 2020-11-06

**Authors:** Dung Anh Nguyen, Son Hong Duong, Phuong Anh Tran, Hai Hoang Cao, Bang Quoc Ho

**Affiliations:** 1 Department of Science and Technology, Ministry of Natural Resources and Environment, 10 Ton That Thuyet Street, My Dinh 2 Ward, Nam Tu Liem District, Hanoi City, Vietnam; 2 Water Resources Institute, 8 Phao Dai Lang Street, Lang Thuong Ward, Dong Da District, Hanoi City, Vietnam; 3 Department of Water Resources Engineering and Technology, Water Resources Institute, 8 Phao Dai Lang Street, Lang Thuong Ward, Dong Da District, Hanoi City, Vietnam; 4 Air Pollution and Climate Change Research Center (APAC), Institute for Environment and Resources (IER), 142 To Hien Thanh, Ward 14, District 10, Ho Chi Minh City, Vietnam; 5 Vietnam National University-Ho Chi Minh City (VNU-HCM), Linh Trung Ward, Thu Duc District, Ho Chi Minh City, Vietnam

**Keywords:** Climate sciences, Environmental sciences

## Abstract

The degradation of air quality is the most concerned issue of our society due to its harmful impacts on human health, especially in cities with rapid urbanization and population growth like Hanoi, the capital of Vietnam. This study aims at developing a new approach that combines data-driven models and interpolation technique to develop the PM_10_ concentration maps from meteorological factors for the central area of Hanoi. Data-driven models that relate the PM_10_ concentration with the meteorological factors at the air quality monitoring stations in the study area were developed using the Multiple Linear Regression (MLR) and Artificial Neural Network (ANN) algorithms. Models’ performance comparison showed that ANN models yielded better goodness-of-fit indices than MLR models at all stations in the study area with average coefficient of correlation (*r*) and Nash–Sutcliffe Efficiency Index (*NSE*) of 0.51 and 0.34 for the former, and 0.7 and 0.49 for the latter. These indices indicates that the ANN-based data-driven models outperformed the MLR-based models. Thus, the ANN-based models and the Inverse Distance Weighting (IDW) interpolation technique were then combined for mapping the monthly PM_10_ concentration with a spatial resolution of 1 km from global meteorological data. With this combination, the PM_10_ concentration maps account for both local PM_10_ concentration and impacts of spatio-temporal variations of meteorological factors on the PM_10_ concentration. This study provides a promising method to predict the PM concentration with a high spatio-temporal resolution from meteorological data.

## Introduction

Air pollution is one of the most concerned issues of our society due to its impacts on human health and the economy^1,2^. Many studies have shown that the mortality rate increases with increasing particulate matter (PM) concentration in the air. For example, Dockery et al.^3^ reported that an increase of 10 μg/m^3^ in the PM_2.5_ concentration would make the death rate increase by 1.5% in 6 cities in the United Stated^4^. A high amount of PM in the air also showed a strong correlation with lung cancer and other cardiovascular and lung related diseases which makes it one of the known causes for deaths in adults in the United States^5^. An increased concentration of 10 μg/m^3^ PM_10_ would result in a 0.5% increase in the number of deaths per day in 20 big cities with more than 50 million inhabitants^6,7^. A similar outcome was also confirmed by Katsouyanni et al.^8^ in a study of over 29 cities. Not only does it have negative effects on human health, PM air pollution also causes huge economic loss. Because of air pollution, each year around 4.1 billion Euro loss was reported in Switzerland^9^ and a loss of 40–50 million USD was revealed by a statistic made by The National Center for Health Statistics of the United States in 2001^10^. According to the World Bank, in 2016 air pollution cost the world’s economy around 5.11 trillion USD in welfare losses. In South Asia and East Asia and the Pacific, losses were around 7.4 percent and 7.5 percent of the regional gross domestic product (GDP), respectively^11^. It has been long recognized that meteorological conditions and PM concentration have a close relationship. Zhao et al.^12^, in a study on PM_2.5_ pollution from 2005 to 2007, highlighted a clear seasonal variation in the concentration of PM_10–2.5_ in which the PM concentration at the rural area was at the minimum level in winter and maximum level in spring and summer, while the urban region experienced the opposite trend. This study also pointed out that precipitation had an important contribution to the seasonal pattern of PM_2.5_ in the urban area, while monsoon was the main factor for that in the countryside. In a similar study, Duo et al.^13^ concluded that temperature in Lhasa, Tibet was the dominant factor governing all air pollutants including PM_10_ and PM_2.5_ in spring, while relative humidity and atmospheric pressure were the major meteorological drivers during summertime. Spatial distribution of fine to coarse PM showed an inverse relationship with wind speed^14,15^. Srimuruganandam and Nagendra^16^ revealed that low wind speed was highly correlated with PM concentration. It was however reported by Giri et al.^17^ that PM_10_ concentration in Kathmandu, Nepal increased with wind speed and atmospheric pressure. Wang and Ogawa^18^ also reported that PM_2.5_ was positively proportional to wind speed higher than 3 m/s, and negatively proportional to the wind speed lower than that level.

Based on the relationship between the PM_10_ concentration and meteorological factors, efforts have been made to construct data-driven models for predicting the PM_10_ concentration from meteorological data using different statistical methods^19–21^. Among these methods, the Multiple Linear Regression (MLR)-based data-driven models are the most popular and have been widely used. The MLR algorithm is usually used to formulate the linear relationship between meteorological factors (including temperature, relative humidity, wind speed, and wind direction) with the PM_10_ or PM_2.5_ concentration. Although there are some studies that reported good prediction results^22^, it is generally seen that the MLR-based models are yet to present consistently satisfactory results due to the linearizing of the non-linear system as reviewed by Shahraiyni and Sodoudi^23^. With the ability to represent complex non-linear problems, the ANN-based data-driven model with different architectures has been extensively used to estimate the PM concentration. Several studies reported that the Artificial Neural Network (ANN) models produced satisfactory prediction results^24^. Although some studies showed that the ANN models performed better than the MLR models^24^, the ANN models have a more complicated structure and still present some limitations in terms of handling high dimensional input variables, local minima or interpretability (the black-box model problem). As a result, for each case study, it is necessary to compare these two models to select the more suitable model.

PM_10_ concentration recorded in air quality monitoring statios only cover an area surrounding those stations, thus it is necessary to use interpolation techniques for mapping the PM_10_ concentration. There are multiple interpolation techniques for this purpose. Wong et al.^25^ provided an excellent review on these techniques and divided them into four groups, namely spatial averaging, nearest neighbor, inverse distance weighting and kriging. These interpolation techniques have been successfully employed to construct PM_10_ maps in many studies. For example, Perez^26^ applied the nearest neighbor technique to provide a forecasting map of PM_10_ in Santiago, Chile. Li et al^27^ developed two IDW-based spatiotemporal interpolation techniques to evaluate the spatial variation of PM_2.5_ concentration over the contiguous United States. Kim et al. employed the ordinary kriging to interpolate the PM_10_ concentration from 226 urban-ambient monitoring sites in South Korea^28^. Raja et al.^29^ used spatiotemporal kriging with the external drift to explore spatio-temporal variations of PM_10_ concentrations in Ankara, Turkey. The main drawback of these studies was that they only used the PM_10_ concentration measured at the air quality stations for interpolation without considering the impacts of the spatio-temporal variations of the meteorological factors on PM_10_ variation. As a result, it is crucial to develop an interpolation technique that can account for information from both air quality stations and meteorological data.

With the increasing population and rapid urbanization, air quality in Vietnam, especially in large cities like Hanoi has been significantly degraded. Hopke et al.^30^ indicated that Hanoi was one of the cities which had the worst air quality in Asia. Saksena et al.^31^ showed that the average value of PM_10_ concentrations in the streets in Hanoi could reach up to 455 μg/m^3^, which is much higher than the Vietnamese daily standard for the PM_10_ concentration (150 μg/m^3^). This has posed a negative effect on the city’s public health. It was reported that ambient and in-house air pollution was becoming the major reason for deaths related to the environment in Vietnam, just second to smoking^32^. As a result, there has been increasing attention and demand from both the local community and the government of Hanoi for a study on air quality and its controlling factors with PM_10–2.5_ concentration prediction being the top priority.

There are two objectives to this study. The first objective is to develop a hybrid mapping approach that combines the data-driven model and IDW interpolation to produce the PM_10_ concentration maps from global meteorological data. The second objective is to employ this approach to construct the monthly PM_10_ maps for the central districts of Hanoi and analyze its spatio-temporal variations.

## Methodology and material

**Figure 1:**
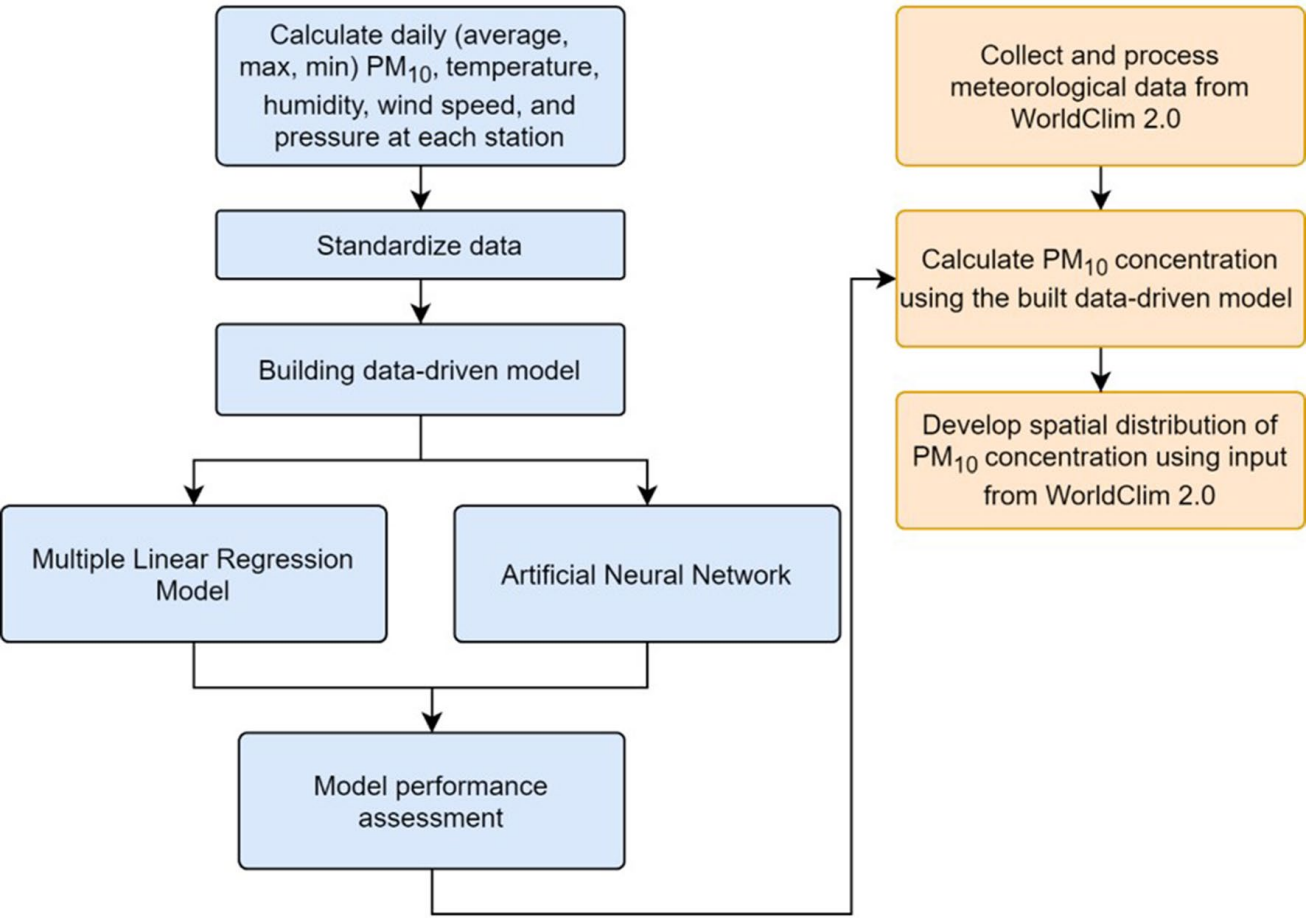
Hybrid data-driven and interpolation scheme.

In this study, we developed a hybrid approach that combines data-driven models and interpolation techniques to construct the PM_10_ concentration maps from global meteorological data. As shown in Fig. 1, this approach consists of two main steps, namely, (1) development of data-driven models at each air quality monitoring station and (2) construction of PM_10_ maps from global meteorological data using the IDW interpolation technique. Details of these two steps are presented below.

### Development of data-driven models

Before developing data-driven models, a set of input features derived from meteorological factors were constructed. After that, the data-driven models linking these selected input features and the PM_10_ concentration using two machine-learning algorithms, namely, MLR and ANN were developed. Finally, based on their performance, the more accurate models were selected for mapping the PM_10_ concentration.

#### Construction of input features

Multiple meteorological factors can be considered in data-driven models to predict the PM_10_ concentration. However, based on the availability of meteorological data at the air quality monitoring stations and their correlation with the PM_10_ concentration, the following variables were taken into account: mean daily temperature, maximum daily temperature, minimum daily temperature, mean daily humidity, mean daily wind speed and mean daily atmospheric pressure. Next, we assumed that the PM_10_ concentration was linked to meteorological factors by a quadratic function as follows:

1 $$PM_{10} = f(X_{i} , \;X_{i}^{2} , \;X_{i} X_{j} )\quad i,\;j = 1, 2, \ldots 6,\;\;i \ne j$$

in which *X*_*i*_ (*i* = 1, 2, …, 6) is the mean daily pressure (*X*_1_), mean daily temperature (*X*_2_), mean daily humidity (*X*_3_), mean daily wind speed (*X*_4_), maximum daily temperature (*X*_5_), minimum daily temperature (*X*_6_). Totally, there are 27 features considered in Eq. (1). Since the size of features is considerably large, features with low correlation coefficients with the PM_10_ concentration or close correlation with other previously-selected features were removed from the equation.

Next, the selected input features and PM_10_ concentration were standardized to avoid the effects of differences in scale of features that could significantly influence the performance of regression models. After standardization, both input features and PM_10_ concentration were unitless values with a mean of 0 and a standard deviation of 1. The standardized features and PM_10_ concentration were used as inputs and outputs for the data-driven models.

#### Development of data-driven models

##### Multiple linear regression model

MLR is a statistical technique used to find a linear relationship between a response variable (dependent variable) and explanatory variables (independent variables). It is one of the most common methods to generalize the relationship of PM concentration with its determinants^23^. Generally, the MLR model is defined by the following equation:

2 $$y_{i} = c_{0} + c_{1} x_{i1} + c_{2} x_{i2} + \cdots + c_{n} x_{in} + \epsilon$$

in which, $y_{i}$ is dependent variable/response variable; $x_{i}$ = independent/explanatory variables; $c_{0}$ is the intercept; $c_{n}$ is the slope coefficient for each independent variables; $\epsilon$ is the error term. The $y_{i}$, in this study, is the standardized PM_10_ concentration, and $x_{i}$ is the standardized features constructed in the previous step. At each air quality monitoring station, a data-driven model based on the MLR algorithm was developed from measurement data of PM_10_ concentration and meteorological-derived input features using the method of least squares.

##### Artificial neural network model

In this study, a feed forward neural network (FFNN) model, a common architecture of ANN model, was employed to build data-driven models for each air quality monitoring station using the same input and output data as in the MLR models for comparison. The structure of a FFNN model consists of three layers (input layer, hidden layer and output layer). We refered to Sanger^33^ for more detailed information about the FFNN algorithm.

In order to develop the FFNN data-driven models, the input and output data were randomly sampled into 3 sub-sets with 70% of data for training, 15% for validation and 15% for testing. Since the number of nodes in the input and output layers was determined, the determination of the FFNN structure focused on determining the number of hidden nodes. In this study, the trial-and-error method was used to find the number of hidden nodes for each air quality monitoring station.

### Development of a hybrid interpolation approach for PM_10_ concentration mapping

Based on the data-driven models developed in the previous section, this study constructed the monthly PM_10_ concentration maps from meteorological data using a new approach based on the IDW interpolation method. In order to predict the PM concentration at a given location (interpolated location) from surrounding air quality monitoring stations, the IDW method determines the weighting factors of each station as below:

3 $$w_{ik} = \frac{{{\raise0.7ex\hbox{$1$} \!\mathord{\left/ {\vphantom {1 {d_{ik}^{2} }}}\right.\kern-\nulldelimiterspace} \!\lower0.7ex\hbox{${d_{ik}^{2} }$}}}}{{\mathop \sum \nolimits_{i = 1}^{N} d_{ik}^{2} }}$$

in which *w*_*ik*_ is the weighting factor of station *i*th at interpolated location *k*th*.* $d_{ik}^{2}$ is the distance from station *i*th to interpolated location *k*th. *N* is the total number of air quality monitoring stations used for interpolation. Using the weighting factors, the PM_10_ concentration was estimated as below:

4 $$PM_{10}^{k} = w_{ik} f_{i}^{{PM_{10} }} ({\varvec{X}}_{k} )$$

in which $f_{i}^{{PM_{10} }}$ is the data-driven model developed for the station *i*th; ${\varvec{X}}_{k}$ is the input feature vector which was derived from meteorological data at interpolated location *k*th. The novel of this approach is that instead of using the PM_10_ concentration values at the air quality monitoring stations like in traditional approaches, it used meteorological data at the interpolated location to feed the data-driven models. This hybrid approach allows us to consider the impacts of both local conditions (via the data-driven model developed for each station) and spatio-temporal variations of meteorological factors.

All the above steps including feature construction, development of MLR and ANN-based models and mapping the PM_10_ concentration were programmed on Matlab (coding of this program is provided in the Supplementary document). This programming language allows for quick implementation of the algorithms and easy visualization of the results without using any other additional software.

The method developed in this study can be well applied for other cities where meteorological and PM_10_ concetration observations are avaiable. However, the selection of meteorological factors and development of data-driven models in this study were purely relied on data from air quality monitoring stations in the city of Hanoi. As a result, the data-driven models may not be applicable to other cities. The data-driven models should be developed for each city based on the availability of observation data in that city.

### Study area

**Figure 2:**
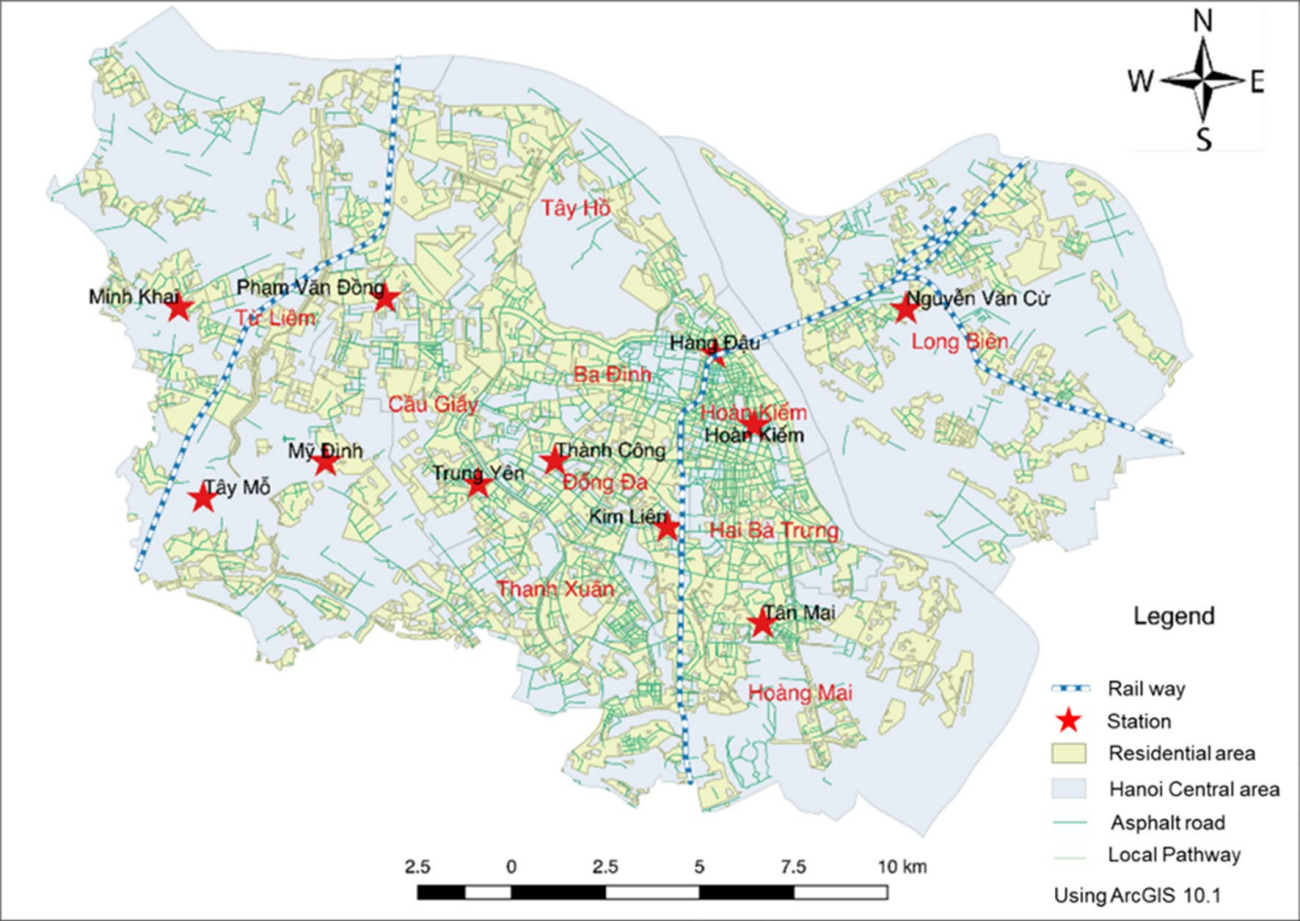
Location of 11 air quality stations used in the study.

**Figure 3:**
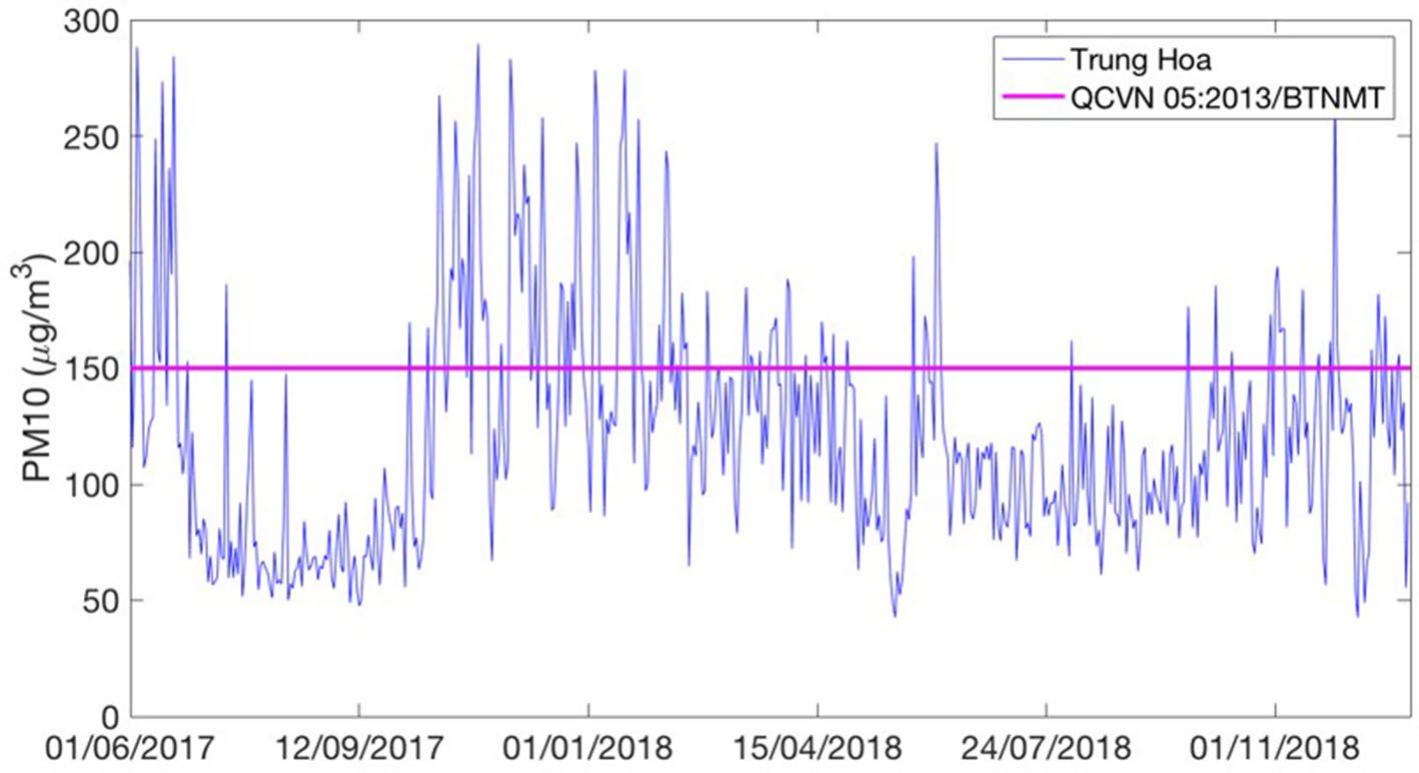
Daily PM_10_ in 2017 in Trung Yen 3 air quality monitoring station.

Hanoi is the capital city of Vietnam with an area of 3358 km^2^ (following the administrative expansion in 2008) and more than 7.4 million people in 2017^34^. The study area consists of eleven central districts of Hanoi (Fig. 2). This is the most crowded area of Hanoi where 41% population of the city resides in 7.7% of the total city area. With a high population density and a large number of vehicles and construction activities, this area has been severely suffered from air pollution. As for the weather conditions, Hanoi has four distinct seasons including spring (March–May), summer (June–August), autumn (September–November) and winter (December–February). In winter, the weather is cold and dry, while summer has high humidity and rainfall^35^. With a lower temperature and low humidity, the PM_10_ concentration in winter is much higher than the other seasons (Fig. 3). While the 24 h PM_10_ concentration is mostly below the National Technical Regulation on Ambient Air Quality (QCVN 05:2013/BTNMT, PM_10_ = 150 μg/m^3^), there are many days in which PM_10_ concentration is above this standard in winter**.** Due to the harmful impacts of high PM_10_ concentration on human health and the importance of the study area, it is necessary to construct high resolution PM_10_ concentration maps for this area in order to provide more detail air quality information for local residents who are most likely to be affected.

### Data availability

The data used in this study was collected from two sources corresponding to two objectives. For the development of the data-driven models, input data was collected from 11 air quality monitoring stations located across the study area (Fig. 2). These stations include three fixed stations (Minh Khai, Trung Yen 3 and Nguyen Van Cu) and eight sensor stations (Table 1). Nguyen Van Cu station is under the management of the Vietnam Environment Administration. The remaining stations are under the management of the Hanoi Department of Environmental Protection. Hourly PM_10_ concentration and meteorological data (atmospheric pressure, temperature, humidity, wind speed) at all stations from 01/06/2017 to 31/12/2018 were collected. This dataset covers one and a haft year, and therefore, can represent the temporal variations of the PM_10_ concentration and meteorological factors over four seasons of the year. In addition to PM_10_, other air pollutants were also collected, although they were not considered in the scope of this study. The hourly PM_10_ concentration and meteorological data were averaged to generate a daily dataset to reduce the measurement errors and remove their diurnal variation.

**Table 1:** Summary information of air quality monitoring stations.

No	Monitoring station	Longitude	Latitude	Site
	**Fixed stations**			
1	Minh Khai	21°02′57.8″	105°44′31.0″	Minh Khai Ward People's Committee
2	Trung Yen 3	21°00′55.1″	105°47′59.6″	Hanoi Department of Environmental Protection
3	Nguyen Van Cu	21°2′58.43″	105°52′55.8″	Northern Center for Environmental monitoring
	**Sensor stations**			
4	Phạm Van Dong	21°03′04.3″	105°46′54.6″	Ha Noi Centre for Environmental and Natural Resources monitoring
5	Tay Mo	21°00′45.2″	105°44′48.1″	Tay Mo Ward People's Committee
6	My Dinh	21°01′10.5″	105°46′13.3″	My Đinh 1 Ward People's Committee
7	Hang Dau	21°02′25.7″	105°50′44.5″	Hang Ma District Police
8	Hoan Kiem	21°01′35.5″	105°51′11.9″	Hoan Kiem District Police
9	Kim Lien	21°00′24.8″	105°50′11.3″	Kim Lien Kindergarten
10	Thanh Cong	21°01′11.0″	105°48′53.0″	Thanh Cong Lake Park
11	Hoang Mai	20°59′18.0″	105°51′17.6″	Hoang Van Thụ Ward People's Committee

**Figure 4:**
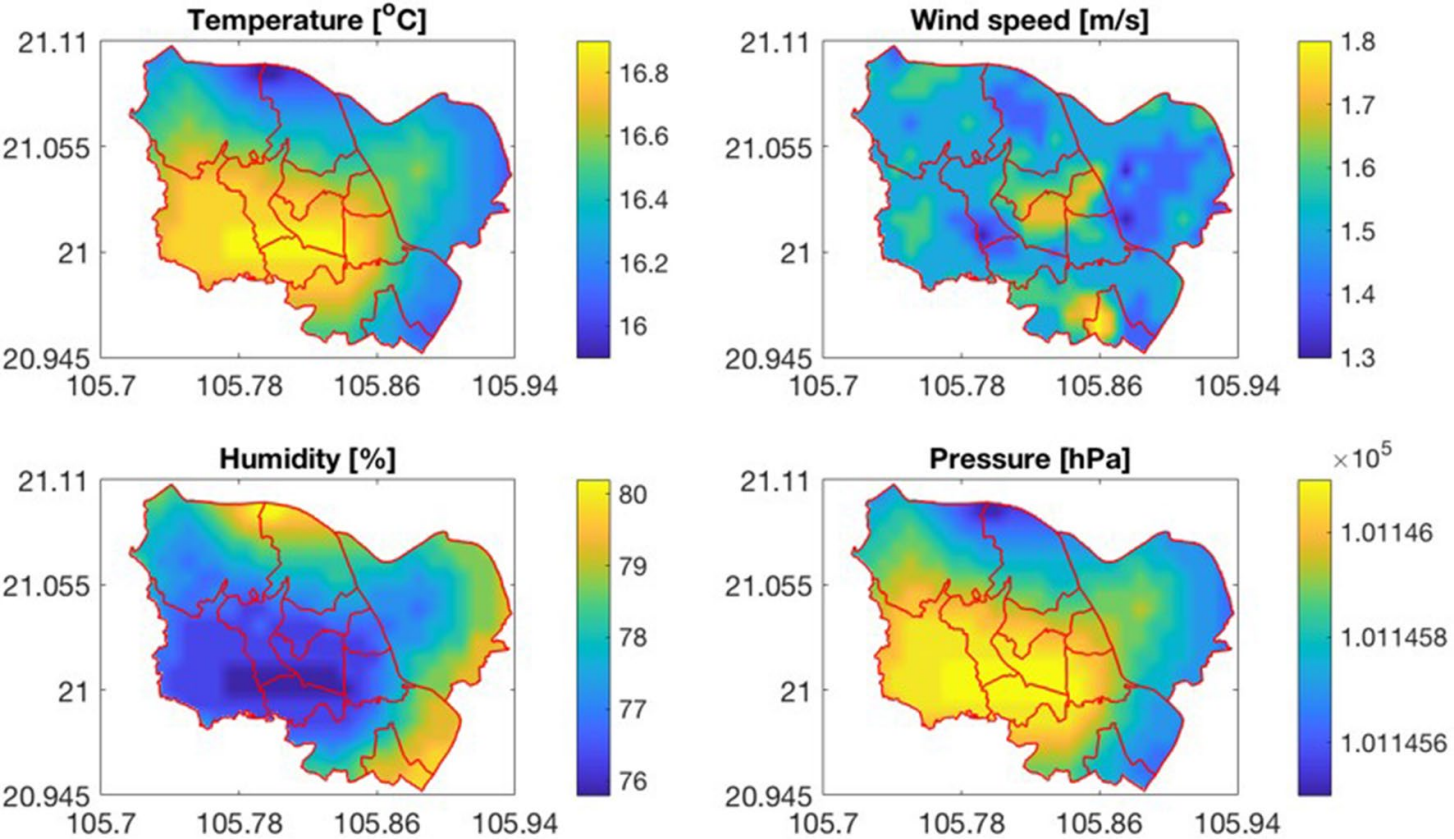
Spatial distribution of Temperature, Humidity, Atmospheric Pressure, and Wind Speed derived from WorldClim 2.0 in January.

Since the data collected from 11 meteorological stations were limited, the PM_10_ concentration calculated from these data was not representative for its spatial variation in the study area. Therefore, high spatial resolution maps of the PM_10_ concentration were needed. For mapping the monthly PM_10_ concentration, we used the global meteorological data from the WorldClim 2.0 database (https://www.worldclim.org/), which contains temperature (mean, maximum, minimum), precipitation, solar radiation, vapor pressure, and wind speed data with a spatial resolution of 1 km^2^. This is a reliable data source that was validated with gauged data (correlation coefficient with gauged data $r \ge 0.99$ for temperature and vapor pressure, $r \ge 0.86$ for precipitation and $r \ge 0.76$ for wind speed). After the global meteorological data was downloaded, they were extracted for the region of the study area. Because the relative humidity was not available, it was calculated from actual and saturated vapor pressure. The atmospheric pressure was calculated from the location altitude and air temperature. Figure 4 shows the temperature, wind speed, relative humidity and air pressure in February obtained from the WorldClim database as an example. As shown in the figure, the data extracted from the WorldClim can well reflect the spatial variation of meteorological factors.

## Results

### Construction of input features for data-driven models

**Figure 5:**
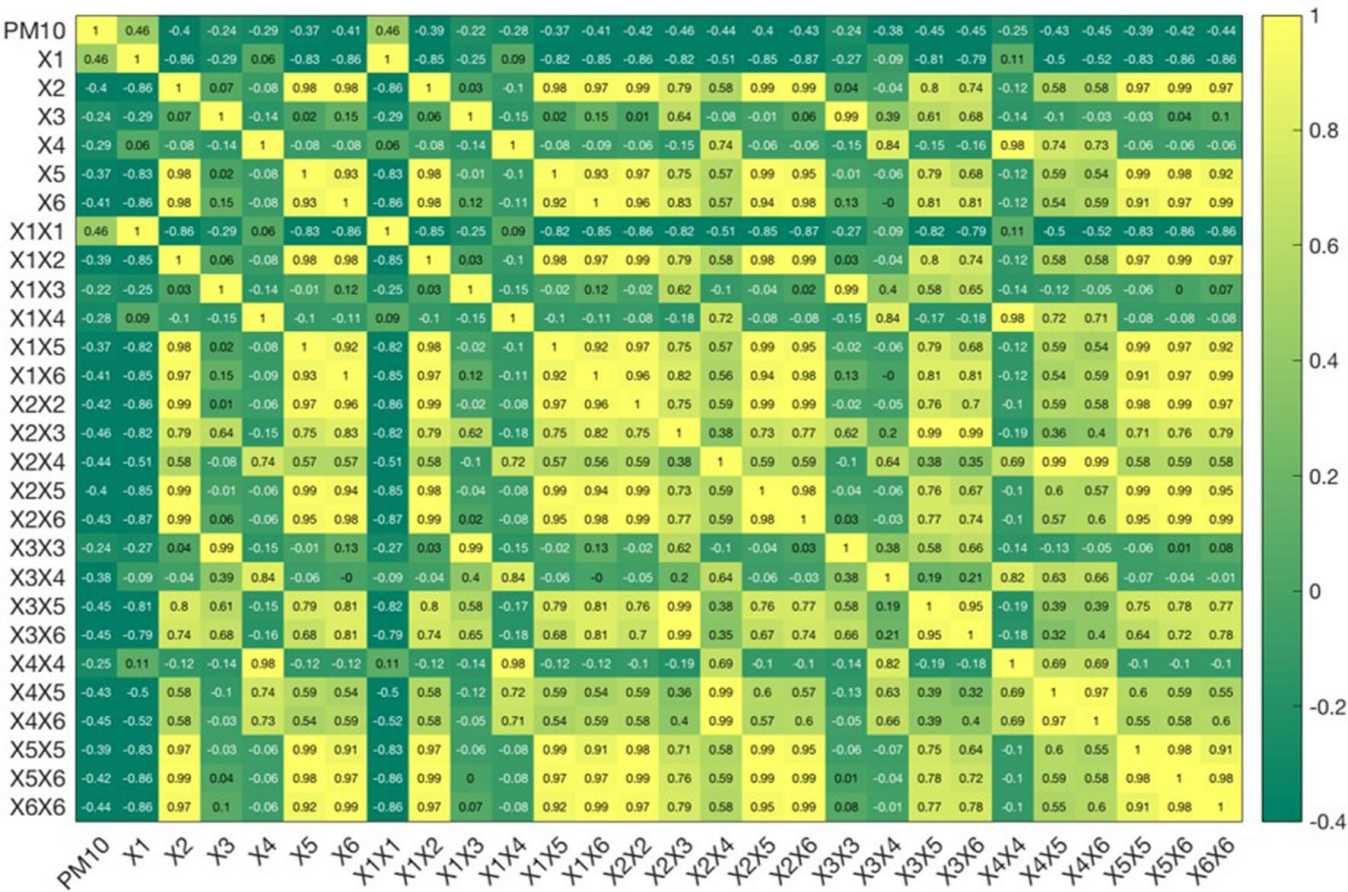
Coerrelation matrix of features and between features with the PM10 concentration. *X*_1_ is the mean daily pressure; *X*_2_ is the mean daily temperature; *X*_3_ is the mean daily humidity; *X*_4_ is the mean daily wind speed; *X*_5_ is the maximum daily temperature; *X*_6_ is the minimum daily temperature.

The meteorological data collected from 01/06/2017 to 31/12/2018 were used to construct the input features for the data-driven models. The total number of features considered in this study was 27. In order to reduce this number of features, the correlation coefficients between each feature with the PM_10_ concentration and between features were estimated. Figure 5 presents the correlation matrix which indicates the correlation coefficients of input features with each other and with the PM_10_ concentration. The figure shows that the correlation coefficients between the input features and the PM_10_ concentration range from − 0.46 to 0.46. All six meteorological factors (mean daily atmospheric pressure, mean daily temperature; mean daily humidity, mean daily wind speed, maximum daily temperature and minimum daily temperature) have a relatively high correlation with the PM_10_ concentration with absolute correlation coefficients greater than 0.24. It is interesting that of these meteorological factors, only the mean daily pressure is positively correlated with the PM_10_ concentration, while the other factors have negative correlation coefficients. This implies that the PM_10_ concentration increases with increasing mean daily pressure and decreasing other factors. The features with the highest correlation with the PM_10_ concentration are the mean daily pressure (*X*_*1*_) and its quadratic term ($X_{1}^{2}$) (correlation coefficients = 0.46), while the product of the mean daily pressure and the mean daily humidity (X_1_X_3_) has the lowest correlation (correlation coefficient = − 0.22).

In order to select input features for the data-driven models, we evaluated the correlation of each feature with the PM_10_ concentration and with the other features. The mean daily pressure (*X*_1_), mean daily temperature (*X*_2_), mean daily humidity (*X*_3_), mean daily wind speed (*X*_4_) are well correlated with the PM_10_ concentration and are independent from each other. As a result, they were added to the input features. The maximum (*X*_5_) and minimum daily temperature (*X*_6_) are well-correlated to the mean daily temperature with correlation coefficients of 0.98. Hence, these two features and their associated features (*X*_1_*X*_5_,* X*_2_*X*_5_,* X*_3_*X*_5_,* X*_4_*X*_5_,* X*_5_*X*_5_,* X*_5_*X*_6_;* X*_1_*X*_6_,* X*_2_*X*_6_, *X*_3_*X*_6_,* X*_4_*X*_6_,* X*_5_*X*_6_,* X*_6_*X*_6_) were not included in the input features. Features *X*_1_*X*_1_*, X*_1_*X*_2_,* X*_1_*X*_3_,* X*_1_*X*_4_,* X*_1_*X*_5_ and *X*_1_*X*_6_ that are functionally correlated with *X*_1_ were not considered either. Of the features associated with the mean daily temperature (*X*_2_) and mean daily humidity (*X*_3_), only features *X*_2_*X*_3_,* X*_2_*X*_4_, and *X*_3_*X*_4_ are relatively independent on the others factors and have a high correlation with the PM_10_ concentration. Therefore, these features were selected as inputs for the data-driven models. In total, the input features consist of *X*_1_*, X*_2_,* X*_3_, *X*_4_*, X*_2_*X*_4_, and *X*_3_*X*_4_.

### Development of data-driven models

#### Multiple linear regression model

Using the input features selected in the previous section, the MLR-based model (Eq. 2) was written as below:

5 $$PM_{10} = c_{1} + c_{2} X_{1} + c_{3} X_{2} + c_{4} X_{3} + c_{5} X_{4} + c_{6} X_{2} X_{3} + c_{7} X_{2} X_{4} + c_{8} X_{3} X_{4}$$

in which the coefficient *c*_*i*_ (*i* = 1…8) was determined from measurement data using the method of least squares. Comparing with previous studies that usually used the MLR algorithm to construct the relationship between the PM_10_ concentration and meteorological factors, this study considered both the meteorological factors (*X*_1_,* X*_2_,* X*_3_, *X*_4_) and their combinative quadratic terms (*X*_2_*X*_3_,* X*_2_*X*_4_,* X*_3_*X*_4_), and therefore, could account for the nonlinear quadratic form of this relationship. In addition, to account for the seasonal dependence of the PM_10_ concentration on meteorological factors, this study built two MLR models corresponding to the winter and spring period and the summer and autumn periods.

#### Artificial neutron network model

**Figure 6:**
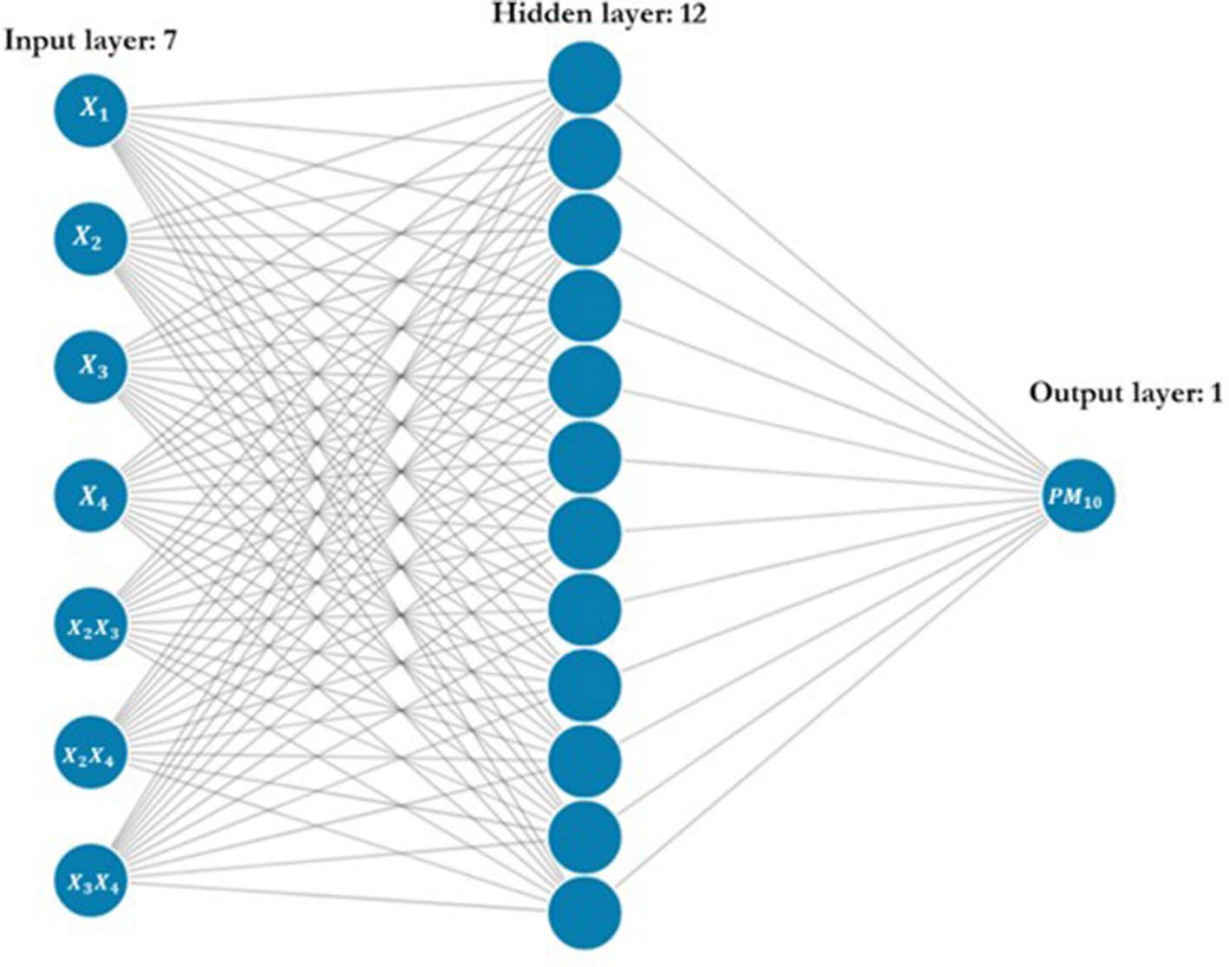
Schematization of the ANN model structure. The model includes 3 layers: input (7 nodes), hidden (12 nodes) and output (1 node).

Although the MLR-based data-driven models developed in this study accounted for the nonlinear and seasonal relationship between the PM_10_ concentration and meteorological factors, they only considered the quadratic nonlinear relationship. For comparison, we employed the ANN algorithm, which could formulate this relationship in a more complicated manner. The ANN model included three layers (input, hidden and output) in which the number of nodes in the input layer and output layer was equal to 7 (number of input features) and 1 (PM_10_), respectively. After using the trial-and-error method, we found that the number of the hidden layers with 12 nodes was the most suitable for all air quality monitoring stations. Figure 6 illustrates the architecture of the ANN model used in this study.

**Figure 7:**
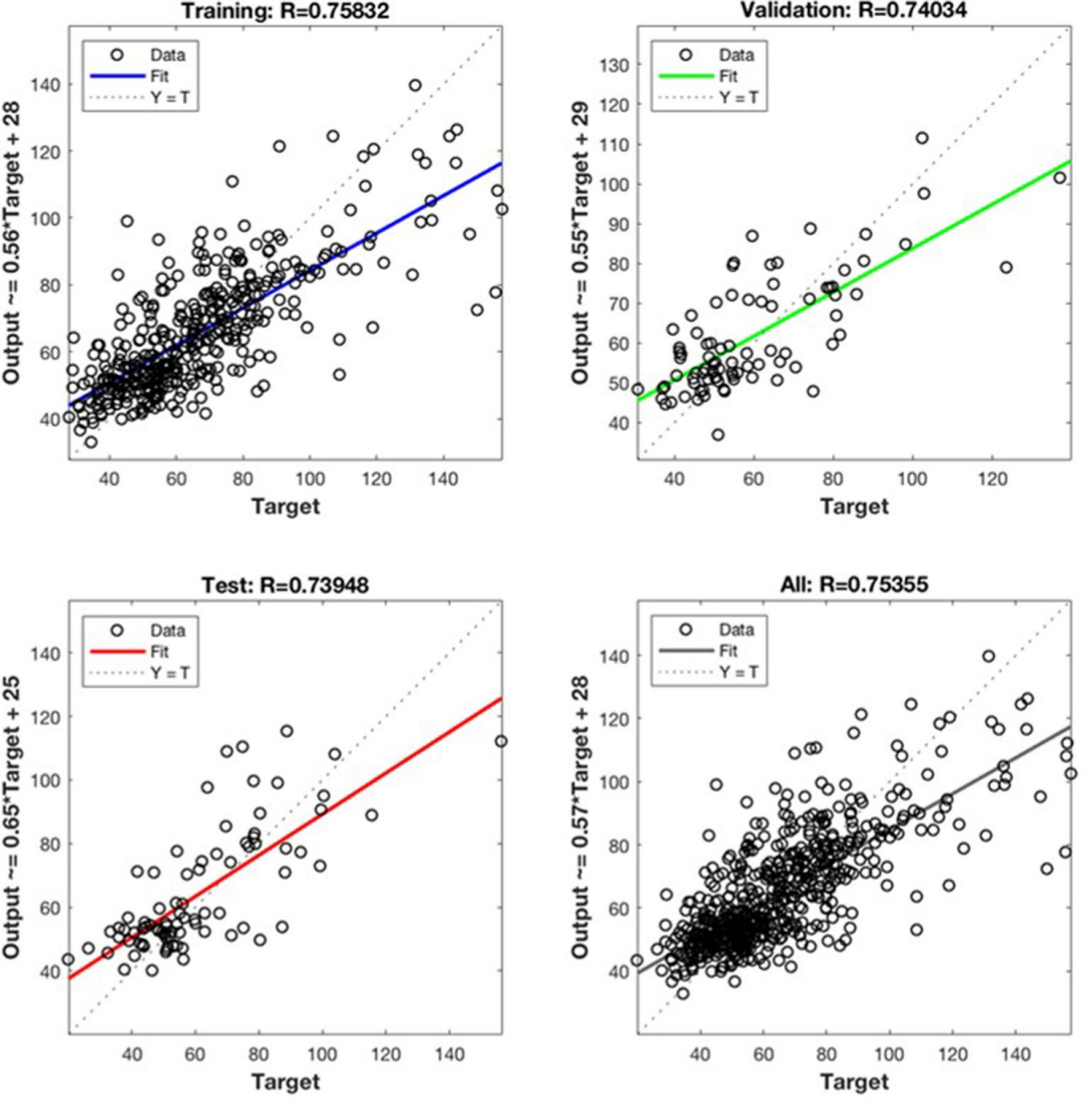
ANN models results for Trung Yen 3 air quality monitoring station.

In order to build the ANN model for each station, the measurement data were divided into three sub-datasets (70% for training, 15% for validation and 15% for testing) to avoid overfitting. Figure 7 compares the PM_10_ concentration between the ANN modeling and measurement in each sub-dataset and the whole dataset at the Trung Yen 3 station as an example. The figure shows that the ANN model could well simulate the PM_10_ concentration at all datasets, and therefore, could be reliably used for predicting PM_10_ concentration.

#### Comparison of model performance

To evaluate the performance of the MLR and ANN models, we used three statistical indices, namely, Root Mean Squared Error (*RMSE*), correlation coefficient (*r*) and Nash–Sutcliffe Efficiency (*NSE*), which are formulated as below:

6 $$r = \frac{{\mathop \sum \nolimits_{i = 1}^{N} \left( {Y_{o}^{i} - \overline{{Y_{o} }} } \right)\left( {Y_{m}^{i} - \overline{{Y_{m} }} } \right)}}{{\sqrt {\mathop \sum \nolimits_{i = 1}^{N} \left( {Y_{o}^{i} - \overline{{Y_{o} }} } \right)^{2} } \sqrt {\mathop \sum \nolimits_{i = 1}^{N} \left( {Y_{m}^{i} - \overline{{Y_{m} }} } \right)^{2} } }}$$

7 $$RMSE = \sqrt {\frac{1}{N}\mathop \sum \limits_{i = 1}^{N} \left( {Y_{m}^{i} - Y_{o}^{i} } \right)^{2} }$$

8 $$NSE = 1 - \frac{{\mathop \sum \nolimits_{i = 1}^{N} \left( {Y_{m}^{i} - Y_{o}^{i} } \right)^{2} }}{{\mathop \sum \nolimits_{i = 1}^{N} \left( {Y_{o}^{i} - \overline{{Y_{o} }} } \right)^{2} }}$$

where *N* is total number of data points; *Y*_*m*_ is the modeled data, *Y*_*o*_ is the observed data. The correlation coefficient ranges from − 1 to 1 in which the higher value corresponds to the closer positive relationship between the modeling and measurement. *RMSE* measures the differences between modeling and measurement. The lower *RMSE* indicates a better agreement between the modeling and measurement. NSE varies in the range from $- \infty$ to 1 in which *NSE* = 1 indicates a perfect match between the modeling and measurement, *NSE* ≤ 0 implies that the model predictions have the same or lower accuracy than the mean of measurements.

Table 2 compares the indices for both data-driven models. The table indicates that the ANN models performed much better than the MLR models in all eleven stations. The ANN outputs are well correlated with the measurement data with an average correlation coefficient of 0.7. No station has a correlation coefficient below 0.65. Meanwhile, the average correlation coefficient of the MLR outputs with measurements is 0.58 in which the Hoan Kiem station has the lowest coefficient (*r* = 0.51). As for the modeling errors, the average *RMSE* of the ANN and MLR models are 14.1 and 15.6, respectively. The *NSE* criterion ranges from 0.41 to 0.57 for the ANN models and from 0.26 to 0.53 for the MLR models. It is clear that the differences between modeling and measurement of the ANN models are lower than those of the MLR models. The reason for this fact is that the ANN algorithm accounts for more complicated interactions between the input and output than the MLR model. For their better performance, the ANN models were employed for mapping the PM_10_ concentration.

**Table 2:** Performance comparison between ANN model and MLR model.

No	Station	ANN model			MLR model		
		*RMSE*	*r*	*NSE*	*RMSE*	*r*	*NSE*
1	Hang Dau	15.86	0.67	0.45	17.15	0.59	0.35
2	Hoan Kiem	9.30	0.65	0.42	10.53	0.51	0.26
3	Kim Lien	9.43	0.70	0.49	10.62	0.60	0.36
4	My Dinh	10.69	0.69	0.47	12.09	0.57	0.32
5	Tuong Mai	9.14	0.69	0.46	10.64	0.52	0.27
6	Thanh Cong	12.49	0.68	0.47	13.91	0.58	0.34
7	Tay Mo	11.98	0.65	0.41	13.37	0.52	0.27
8	Minh Khai	24.03	0.72	0.51	26.08	0.60	0.36
9	Trung Yen 3	15.69	0.75	0.56	16.24	0.73	0.53
10	Nguyen Van Cu	15.72	0.75	0.57	19.21	0.60	0.35
11	Pham Van Dong	20.19	0.73	0.53	21.96	0.61	0.37

Of all air quality monitoring stations, the performance of both MLR and ANN at the Trung Yen 3 3, Nguyen Van Cu and Minh Khai stations are much better than the others. Indeed, the average correlation coefficient and NSE corresponding to the ANN models for the three stations are respectively equal to 0.74 and 0.55, while these indices for the other stations are much lower (0.68 for the correlation coefficient and 0.46 for the NSE). This could be explained by the fact that Trung Yen 3, Nguyen Van Cu, Minh Khai are the main stations of Hanoi, which have been frequently checked and performed quality control. As a result, the quality of measurement data at these stations is better than the others.

### Monthly PM_10_ concentration mapping

**Figure 8:**
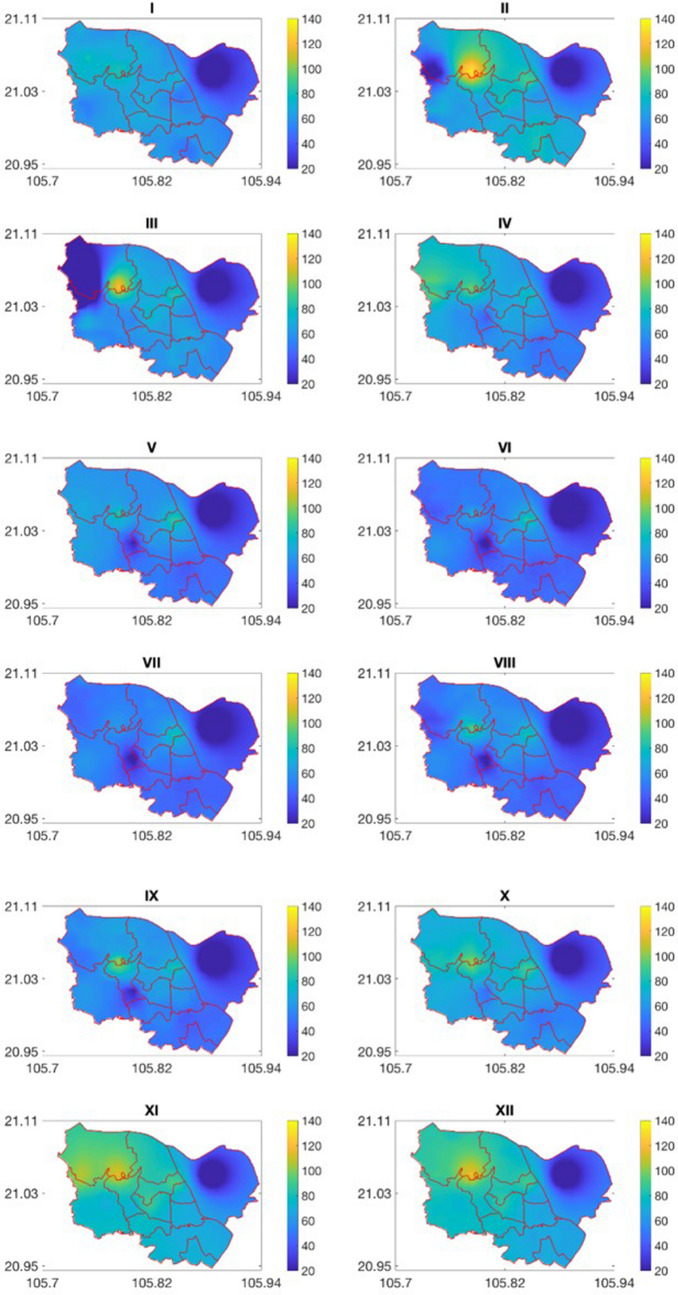
Monthly maps of PM_10_ concentration.

**Figure 9:**
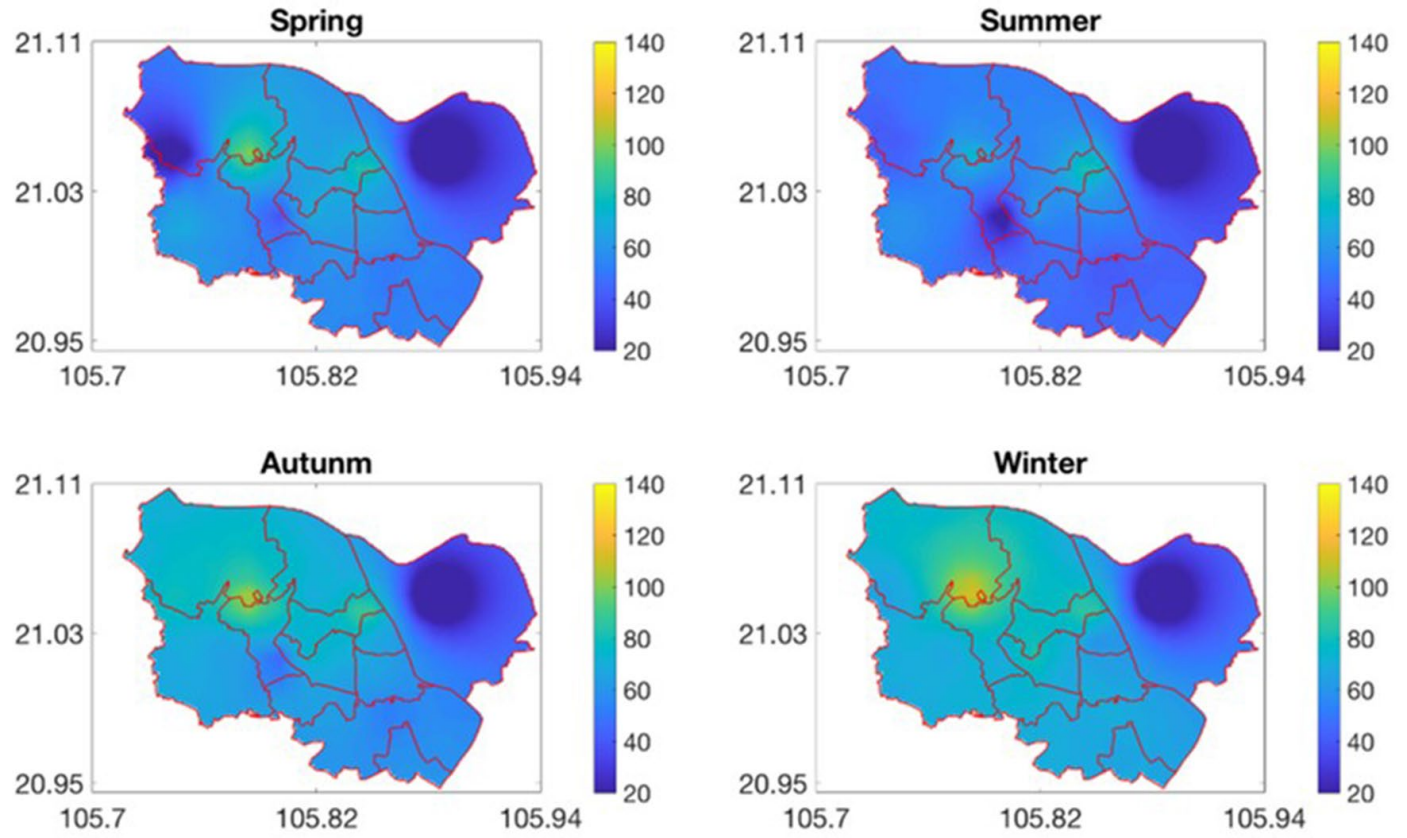
Seasonal maps of the PM10 concentration. The red-solid lines represent the administrative boundary of eleven districts.

Mapping the monthly PM_10_ concentration for the study area of eleven central districts in Hanoi from the WorldClim global meteorological data was performed using the ANN models developed in the previous section and the hybrid interpolation approach (Eq. 4). Figures 8 and 9 below presents the monthly and seasonal maps of the PM_10_ concentration. The spatial resolution of these maps is equal to that of meteorological data (1 km^2^). The seasonal PM_10_ concentration maps were generated by assembling monthly maps.

As for the temporal variation of the PM_10_ concentration, it can be seen that the PM_10_ concentration reaches its peaks in the winter season (January, February, November and December). For example, the mean PM_10_ concentration in November is up to 71 μg/m^3^. The low temperature (ranging from 16 to 21 °C in these months) and the temperature inversion phenomenon in winter are likely the causes of the high PM_10_ concentration in these months. By contrast, because the air temperature in June–August reaches its highest level (~ 29 °C), the concentration of PM_10_ hits a trough in this period with the PM_10_ concentration ranging from 46 to 48 μg/m^3^ As regards to the seasonal variation, points out that the PM_10_ concentration is highest in winter and lowest in summer. In addition to the air temperature, the humidity and wind speed, which are lowest in winter, are also the reason for the higher PM_10_ concentration in winter than in the other seasons.

As for the spatial variation, the concentration of PM_10_ in Long Bien district which is situated in the northeast of the study area is much lower than the other districts. The main reason is that compared to the other districts in the study area, the density of population in this district is lowest in the study area (around 4.5 thousand people/km^2^, versus 11.6 thousand people/km^2^ in other districts). This lower population density leads to less intensive traffic. Besides, due to the sparse air quality monitoring network, the PM_10_ concentration in Long Bien district strongly depends on the PM_10_ concentration at the Nguyen Van Cu station, which situates relatively far away from transportation routes. On the other hand, as pointed out by Nghiem et al.^36^, since the inauguration of Vinh Tuy Bridge in 2010 and Nhat Tan Bridge in 2015, the flow of traffic vehicles through Nguyen Van Cu road was decreased. As a result, the annual average of PM_10_ concentration at Nguyen Van Cu station from 2010 to 2018 slightly declined which makes the PM_10_ concentration lower. On the contrary, the highest concentration is found at the Pham Van Dong station located in the southwest of the study area. This station is placed on the Pham Van Dong Street, one of the main route to access Hanoi from Noi Bai International Airport, thus the traffic in this street is normally quite intensive. Besides, a high number of active construction works in this area might be an important factor for the increased level of PM_10_. Meteorological conditions also influence the spatial distribution of the PM_10_ concentration. The highest PM_10_ concentration in the northwest region is partly caused by the low air temperature in this region (see Fig. 4). However, Fig. 8 shows that the impact of local factors (e.g., street, population, transportation intensity) on the spatial variation of the PM_10_ concentration is larger than that of meteorological factors.

## Conclusion

In this study, a combinative approach of data-driven models and IDW interpolation technique was developed to construct the PM_10_ concentration maps for the central area of Hanoi. The construction of data-driven models consisted of two steps, feature construction and model development. The feature construction is responsible for constructing optimal features from meteorological factors. By evaluating the correlation between the PM_10_ concentration with each feature and correlation between features, a set of features was selected as the input for the data-driven models. The model development step built the data-driven models that link the PM_10_ concentration with the input features using the MLR and ANN algorithms for each air quality monitoring station. The obtained results indicate that the ANN-based data-driven models provided much better results than the MRL-based models. In order to construct the PM_10_ concentration maps, the IDW interpolation technique was used to calculate the weighting factors for each air quality monitoring stations. While many other studies obtained the unknown PM_10_ concentration by interpolating the PM_10_ concentration at the air quality monitoring stations without considering meteorological factors, this study accounted for the meteorological factors in the data-driven models. Using this approach, both the local PM_10_ surrounding monitoring stations and the dependence of PM_10_ on meteorological factors were taken into account hence provided a better representation of the current situation in the study area.

Due to a lack of high spatial resolution of meteorological data, this study used the 1 km^2^ resolution monthly WorldClim data as the input to predict monthly PM_10_ concentration via combination of the established data-driven models and interpolation method. The monthly PM_10_ maps were then aggregated to construct seasonal maps. The temporal analysis revealed that the PM_10_ concentration was highest in the winter months and lowest in the summer months, which was mainly caused by the negative dependence of the PM_10_ concentration on air temperature and low humidity. The spatial analysis indicated that the northeast region was the region with the lowest PM_10_ concentration because the urbanization in this region was less developed than the others. The northwest region had the highest PM_10_ concentration because of the high population and ongoing constructions of new buildings and roads, which together elevated the PM_10_ concentration. The meteorological factors also influenced the spatial variation of the PM_10_ concentration but with a lower impact level compared to the local sources of PM_10_ generation surrounding the monitoring stations. This study also pointed out that although the spatial variation of meteorological factors was taken into account, the low density of air quality monitoring stations might reduce the accuracy of PM_10_ concentration maps. Hence, it is necessary to establish a denser air quality monitoring stations network to better cover the spatial variation of the PM_10_ concentration. The approach developed in this study can be applied to provide the forecasting PM_10_ concentration maps based on predicting meteorological information. These results could also provide a very meaningful foundation for the local authority in deriving and implementing city air quality management activities and urban planning in Hanoi.

## Supplementary information

Supplementary Information.
