# Supplementary material for: Combination of data-driven models and interpolation technique to develop of PM10 map for Hanoi, Vietnam
Source: Sci Rep. 2020 Nov 6;10:19268. doi: 10.1038/s41598-020-75547-y (PMC7648064; doi:10.1038/s41598-020-75547-y)
Supplement: Supplementary file 1 — Supplementary Information. [file 41598_2020_75547_MOESM1_ESM.docx]

**Combination of Data-driven Models and Interpolation Technique to Develop of PM10 Map for Hanoi, Vietnam**

**Nguyen Anh Dung, Duong Hong Son, Tran Anh Phuong, Cao Hoang Hai, Ho Quoc Bang**

**apply_AI_Model**

close all;clear all;clc;

%[x,t] = simplefit_dataset;

%errors=ANN(x,t);

%return

write_to_csv_file='n';

plot_histogram='n';

%% Consideration factor

symbollib=['x','o','s','v','x','o','s','v','x','o','s','v','x','o','s','v'];

colorlib=['r','b','m','c','b','m','y','r','m','c','b','m','y','r'];

XName={'Daily mean PM10 [\mug/m^3]','Daily mean P [hPa]','Daily mean T [^oC]','Daily mean RH [%]','Daily mean Wind speed [m/s]','Daily max P [hPa]','Daily max T [^oC]','Daily max RH [%]','Daily max Wind speed [m/s]','Daily min P [hPa]','Daily min T [^oC]','Daily min RH [%]','Daily min Wind speed [m/s]'};

filenames={'1HDFilterData';'2HKFilterData';'3KLFilterData';'4MDFilterData';'5PVDFilterData';'6TMaiFilterData';'7TCFilterData';'8TMoFilterData';'MKFilterData';'ThoaFilterData';'NVCFilterData'};

stationName={'Hang Dau','Hoan Kiem','Kim Lien','My Dinh','Pham Van Dong','Tuong Mai','Thanh Cong','Tay Mo','Minh Khai','Trung Hoa','Nguyen Van Cu'};

for k=10

filename=filenames{k};

if exist([filename '_NN.mat'])~=2

if strcmp(filename,'MKFilterData') || strcmp(filename,'ThoaFilterData') || strcmp(filename,'NVCFilterData')

factorIndex=[2,3,4,7];

else

factorIndex=[2,3,4,5];

end

[X,XName,y,meanX,stdX,meany,stdy]= create_features(filename,factorIndex,'n');

%% Filter X

X=X(:,[1,2,3,4,14,15,19]);

XName=XName([1,2,3,4,14,15,19]);

meanX=meanX([1,2,3,4,14,15,19]);

stdX=stdX([1,2,3,4,14,15,19]);

inputs=X';

targets=y';

rAll=-999;

rTrain=-999;

rTest=-999;

while rAll<0.745 || rTest<0.71 || rVal<0.71

[outputs,net,rVal,rAll,rTrain,rTest]=ANN(inputs,targets);

rAll

rTrain

rTest

end

save([filenames{k} '_NN'],'net','outputs','rVal','rAll','rTrain','rTest');

else

if strcmp(filename,'MKFilterData') || strcmp(filename,'ThoaFilterData') || strcmp(filename,'NVCFilterData')

factorIndex=[2,3,4,7];

else

factorIndex=[2,3,4,5];

end

[X,XName,y,~,~,~,~]= create_features(filename,factorIndex,'n');

X=X(:,[1,2,3,4,14,15,19]);

maxV=max(X(:,[2,3,4]),[],1);

XName=XName([1,2,3,4,14,15,19]);

targets=y';

load([filenames{k} '_NN'],'net','outputs','rVal','rAll','rTrain','rTest');

save([filenames{k} '_NN'],'net','outputs','rVal','rAll','rTrain','rTest','maxV');

[bias(k),rmse(k),corr(k),nash(k)]=stat_criteria(outputs,targets);

end

%% Compare Model and measurement

figure; hold on;box on;

plot(targets,outputs,'ok');

plot([0,160],[0,160],'r');

xlim([0,160]);

ylim([0,160]);

xlabel('Observed PM10 [\muy/m^3]');

ylabel('Modeled PM10 [\muy/m^3]');

set(gca,'fontsize',14);

end

return

%%

%% Plot histogram

if strcmp(plot_histogram,'y')==1

figure;

for k=1:size(X,2)

subplot(ceil(size(X,2)/3),3,k);

histogram(X(:,k));

set(gca,'fontsize',12);

title(XName{k});

end

end

%% Write to csv file

if strcmp(write_to_csv_file,'y')==1

fid=fopen(['So lieu 11 tram_2018/Daily_' filename '.csv'],'wt');

fprintf(fid,'y, X\r\n');

for j=1:size(X,1)

fprintf(fid,'%f %s',y(j),',');

for k=1:size(X,2)

fprintf(fid,'%f%s',X(j,k),',');

end

fprintf(fid,'\r\n ');

end

fclose(fid);

end

**EstimatePMFromWorldClim_AI**

close all;clear all;clc;

innerHanoi=shaperead('/Users/aptran/Documents/VienTNN/Research/LATS_Dung/Data/Hanoi/HanoiComune');

filenames={'1HDFilterData';'2HKFilterData';'3KLFilterData';'4MDFilterData';'5PVDFilterData';'6TMaiFilterData';'7TCFilterData';'8TMoFilterData';'MKFilterData';'ThoaFilterData';'NVCFilterData'};

monthName={'Thang: I','Thang: II','Thang: III','Thang: IV','Thang: V','Thang: VI','Thang: VII','Thang: VIII','Thang: IX','Thang: X','Thang: XI','Thang: XII'};

monthName2={'I','II','III','IV','V','VI','VII','VIII','IX','X','XI','XII'};

%% Location of weather stations

YLoc(11)=21.04905; XLoc(11)=105.88264;

YLoc(9)=21.04938889; XLoc(9)=105.7419444;

YLoc(10)=21.01530556; XLoc(10)=105.7998889;

YLoc(5)=21.05119444; XLoc(5)=105.7818333;

YLoc(8)=21.01255556; XLoc(8)=105.7466944;

YLoc(4)=21.01958333; XLoc(4)=105.7703611;

YLoc(1)=21.04047222; XLoc(1)=105.8456944;

YLoc(2)=21.02652778; XLoc(2)=105.8533056;

YLoc(3)=21.00688889; XLoc(3)=105.8364722;

YLoc(7)=21.01972222; XLoc(7)=105.8147222;

YLoc(6)=20.98833333; XLoc(6)=105.8548889;

%% Get coefficient

if exist('betaCoef.mat','file')~=2

filename={'THoaFilterData';'MKFilterData';'NVCFilterData'};

for k=1:length(filename)

[class_HighLow_beta{k},~,~]= MultiVariate_Model(filename{k});

end

save('betaCoef','class_HighLow_beta');

else

load('betaCoef','class_HighLow_beta');

end

%% Load world clim data and refine data

load WorldClim xpm ypm gridData;

[refinedX,refinedY]=meshgrid(linspace(105.7,105.94,250),linspace(20.945,21.11,180));

XtickValue=(linspace(min(refinedX(:)),max(refinedX(:)),4));

YtickValue=(linspace(min(refinedY(:)),max(refinedY(:)),4));

varName={'Temperature [^oC]','Wind speed [m/s]','','Humidity [%]','Pressure [hPa]'};

%% Calculate Pressure from T

for k=1:12

gridData{5}{k}=atmPressure(gridData{1}{k});

end

%% Filter the region

watershedBound='/Users/aptran/Documents/VienTNN/Research/LATS_Dung/Data/Hanoi/innerHanoi1region';

SRiver = shaperead(watershedBound);

Xbound=SRiver.X';

Ybound=SRiver.Y';

refinedX1D=reshape(refinedX,[],1);

refinedY1D=reshape(refinedY,[],1);

RegionID1D = inpolygon(refinedX1D,refinedY1D,Xbound,Ybound);

RegionID=reshape(RegionID1D,size(refinedX,1),size(refinedX,2));

%% Plot meteorological data

figure('Position',[0,0,800,450]); hold on;box on;

imonth=7;

idraw=1;

for k=[1,2,4,5]

subplot(2,2,idraw);

temp=interp2(xpm,ypm,gridData{k}{imonth},refinedX,refinedY);

temp(RegionID==0)=nan;

plot2DMap(refinedX,refinedY,temp,XtickValue,YtickValue,varName{k},'pcolor');hold on;

for iRegion=1:length(innerHanoi)

mapshow(innerHanoi(iRegion).X,innerHanoi(iRegion).Y,'LineWidth',1,'Color','r');

end

% mapshow(SRiver.X,SRiver.Y,'LineWidth',1,'Color','r');

%mapshow('/Users/aptran/Documents/VienTNN/Research/LATS_Dung/Data/Hanoi/duongnhua.shp','Color','yellow');

%mapshow('/Users/aptran/Documents/VienTNN/Research/LATS_Dung/Data/Hanoi/duongsat.shp','Color','yellow','linewidth',1.5,'LineStyle','--');

idraw=idraw+1;

end

%% Calculate weights

col=1;

for k=1:size(xpm,1)

for j=1:size(xpm,2)

dis=(xpm(k,j)-XLoc).^2 + (ypm(k,j)-YLoc).^2;

w(col,:)=(1./dis)/sum(1./dis);

col=col+1;

id(k,j)=find(dis==min(dis));

end

end

%% Calculate PM10

for month=1:12

clear X

col=1;

for k=1:size(xpm,1)

for j=1:size(xpm,2)

clear temp

temp=[gridData{5}{month}(k,j),gridData{1}{month}(k,j),gridData{4}{month}(k,j),gridData{2}{month}(k,j)]' ;

X(:,col)=[temp; temp(2)*temp(3);temp(2)*temp(4);temp(3)*temp(4)];

col=col+1;

end

end

%

PM10Component=nan(size(w));

for h=1:length(filenames)

clear temp

load([filenames{h} '_NN'],'net','maxV');

temp=[X(1:4,:); X(2,:).*X(3,:)/(maxV(1)*maxV(2));X(2,:).*X(4,:)/(maxV(1)*maxV(3));X(3,:).*X(4,:)/(maxV(2)*maxV(3))];

% PM10Component=net(temp);

PM10Component(:,h)=net(temp);

end

% X=999.2882 31.0353 62.5765 1.7647 0.5907 0.4778 0.3911

% Calculate PM10

col=1;

for k=1:size(xpm,1)

for j=1:size(xpm,2)

% PM10{month}(k,j)=PM10Component(col);%*w(col,:)';

PM10(month,k,j)=PM10Component(col,:)*w(col,:)';

col=col+1;

end

end

end

%% Calculate area mean

% For meteorological data

for ifactor=1:5

for imonth=1:12

temp=gridData{ifactor}{imonth};

areaMeanMeteo(ifactor,imonth)=mean(temp(:));

end

figure;

plot([1:12],areaMeanMeteo(ifactor,:),'*-');

end

%% For PM10

for month=1:12

clear temp

temp=squeeze(PM10(month,:,:));

AreaMean(month)=mean(temp(:));

end

figure;hold on;box on;

plot([1:12],AreaMean,'ob-');

set(gca,'fontsize',14,'Xtick',[1:12],'Xticklabel',monthName2);

ylabel('PM10 [mug/m^3]');

%% Calculate yearly-mean, season-mean

%M?a t?nh theo kh? t??ng d?a v?o nhi?t ??, m?a xu?n t? 1/3 ??n 31/5, m?a h? t? 1/6 ??n 31/8, m?a thu t? 1/9 ??n 31/11 v? m?a ??ng t? 1/12 ??n h?t th?ng 2.

yearMean=squeeze(mean(PM10,1));

seasonMean(1,:,:)=squeeze(mean(PM10(3:5,:,:),1));

seasonMean(2,:,:)=squeeze(mean(PM10(6:8,:,:),1));

seasonMean(3,:,:)=squeeze(mean(PM10(9:11,:,:),1));

seasonMean(4,:,:)=(squeeze(PM10(12,:,:)) + squeeze(mean(PM10(1:2,:,:),1)))/2;

seasonMean(5,:,:)=squeeze(mean(PM10(1:12,:,:),1));

seasonName={'Mua xuan','Mua He','Mua thu','Mua Dong','Trung binh nam'};

%seasonName={'Spring','Summer','Autunm','Winter','Annual'};

%% Find region with above and below

standardMap=nan(size(seasonMean));

for k=1:size(seasonMean,1)

temp=squeeze(seasonMean(k,:,:));

temp(temp<=25)=0.5;

temp(temp>25 & temp<=50)=1.5;

temp(temp>50 & temp<=75)=2.5;

temp(temp>75)=3.5;

standardMap(k,:,:)=temp;

end

%% Calculate AQI

for k=1:size(PM10,1)

temp=squeeze(PM10(k,:,:));

for i=1:size(temp,1)

for j=1:size(temp,2)

AQIMap(k,i,j)=AQI(temp(i,j));

end

end

end

%% Plot PM10

plotPM10(SRiver,innerHanoi,xpm,ypm,refinedX,refinedY,XtickValue,YtickValue,RegionID,3,seasonName,standardMap,[0,4]);

return

%% Plot above or below standard

plotPM10(SRiver,innerHanoi,xpm,ypm,refinedX,refinedY,XtickValue,YtickValue,RegionID,7,monthName,AQIMap,[1,5]);

%% Calculate different

seasonDiff=nan(size(seasonMean));

for k=1:4

seasonDiff(k,:,:)=squeeze(seasonMean(k,:,:))-yearMean;

end

%% Plot data

k=1;

month=9;

plotPM10(SRiver,innerHanoi,xpm,ypm,refinedX,refinedY,XtickValue,YtickValue,RegionID,month,monthName2,PM10);
